# Data on the crystal structures of β-glucosidase from *Thermoanaerobacterium saccharolyticum*

**DOI:** 10.1016/j.dib.2024.111019

**Published:** 2024-10-15

**Authors:** Ki Hyun Nam

**Affiliations:** College of General Education, Kookmin University, Seoul 02707, Republic of Korea

**Keywords:** β-glucosidase (Bgl), Loop structure, Structural dynamics, Temperature factor

## Abstract

β-Glucosidase (Bgl) is a biomass-degrading enzyme that hydrolyzes cellobiose and glucose-substituted polysaccharides into glucose, playing a crucial role in enzymatic saccharification during biofuel production. Despite the wealth of structural information available on Bgl, the molecular properties of the loops above the substrate-binding pocket remain unexplored. In previous study, to better understand the molecular functions of these loop regions, four crystal structures of *Thermoanaerobacterium saccharolyticum* Bgl (TsaBgl) were determined. The molecular flexibility and conformational changes of the loop regions in TsaBgl were analysed, expanding our understanding of their roles in the Bgl family. The data processing and structure determination details provided here are valuable for further studies on the structural properties of these loop regions.

Specifications Table

**Table:** 

Subject	Biological science
Specific subject area	Structural Biology
Type of data	Processed
Data collection	Synchrotron: Pohang Light Source II (PLS-II) Beamline: 11C Detector: Pilatus 6M X-ray energy: 12.569 keV Data collection temperature: 100 K Data processing program: HKL2000
Data source location	Institution: Kookmin University City/Town/Region: Seoul Country: Republic of Korea
Data accessibility	**X-ray diffraction images** Repository name: Zenodo Data identification number: 10.5281/zenodo.8424379 Direct URL to data: https://zenodo.org/records/8424379 **Structure factor and coordinates** Repository name: Protein Data Bank Data identification number: Data I: 10.2210/pdb8WFT/pdb Data II: 10.2210/pdb8WFU/pdb Data III: 10.2210/pdb8WFV/pdb Data IV: 10.2210/pdb8WFW/pdb Direct URL to data: Data I: https://www.rcsb.org/structure/8WFT Data II: https://www.rcsb.org/structure/8WFU Data III: https://www.rcsb.org/structure/8WFV Data IV: https://www.rcsb.org/structure/8WFW Instructions for accessing these data: X-ray diffraction images, coordinate and structure factor can be download without permission.
Related research article	K.H. Nam, The Conformational Change of the L3 Loop Affects the Structural Changes in the Substrate Binding Pocket Entrance of β-Glucosidase. Molecules 2023, 28(23), 7807; https://doi.org/10.3390/molecules28237807 [1].

## 1 Value of the Data

• Four crystal structures of β-glucosidases from *Thermoanaerobacterium saccharolyticum* were determined.
• The data provide coordinates and structure factors for the crystal structures of TsaBgl.
• The data reveal two distinct conformations of the L3 loop region in TsaBgl, indicating variability under different crystallization conditions.
• The findings show that the L3 loop exhibits greater flexibility compared to other loop regions in TsaBgl.
• These data offered insights into the structural dynamics of loop regions in the Bgl family.

## 2 Background

β-Glucosidases (Bgls) hydrolyze β-1,4-glycosidic bonds in cellobiose and glucose-substituted polysaccharides [[2], [3], [4], [5]]. Bgls are used in various biotechnological processes, including the production of carbohydrate-based foods and other commercial products [[5], [6], [7]]. This enzyme is applied in ethanol and biofuel production from biomass [[8], [9], [10]]. In Bgl crystal structures, four loops above the substrate-binding pocket form the entrance to this pocket and display unique conformations, suggesting that these loops may change during enzyme reactions [3,11]. However, the structural and conformational characteristics of these loops remain incompletely understood. Previous research on the Bgl crystal structure from *Thermoanaerobacterium saccharolyticum* (TsaBgl) provided insights into the conformational changes and conservation of loops among Bgls. To enhance the use of this dataset and the structural understanding of TsaBgl, detailed data on the collection, structure determination, and analysis of TsaBgl are reported.

## 3 Data Description

Crystallography experiments for TsaBgl were conducted, resulting in four X-ray diffraction (XRD) datasets named Data I–IV. Data I and II crystals were grown at pH 7.5, while Data III and IV crystals were grown at pH 8.0. The datasets for Data I–IV were processed using 360, 360, 200, and 140 XRD images, respectively. Indexing of XRD images indicated that Data I and II belong to the triclinic P1 space group, with unit cell dimensions of Data I (*a* = 63.414 Å, *b* = 72.831 Å, *c* = 97.490 Å, α = 92.495°, β = 91.281°, and γ = 95.128°) and Data II (*a* = 63.213 Å, *b* = 72.739 Å, *c* = 97.385 Å, α = 92.484°, β = 91.431°, and γ = 95.215°). Matthews coefficient calculations for Data I and II indicated four molecules in the asymmetric unit, with a Vm of 2.12 Å^3^/Da and solvent contents of 41.89 %. Data III and IV belong to the orthorhombic P2_1_2_1_2_1_ space group, with unit cell dimensions for Data III (*a* = 64.970 Å, *b* = 70.948 Å, and *c* = 98.876 Å) and Data IV (*a* = 65.066 Å, *b* = 71.211 Å, and *c* = 99.151 Å). Matthews coefficient calculations for Data III and IV indicated two molecules in the asymmetric unit, with a Vm of 2.21 Å^3^/Da and solvent contents of 44.30 %.

**Fig. 1:**
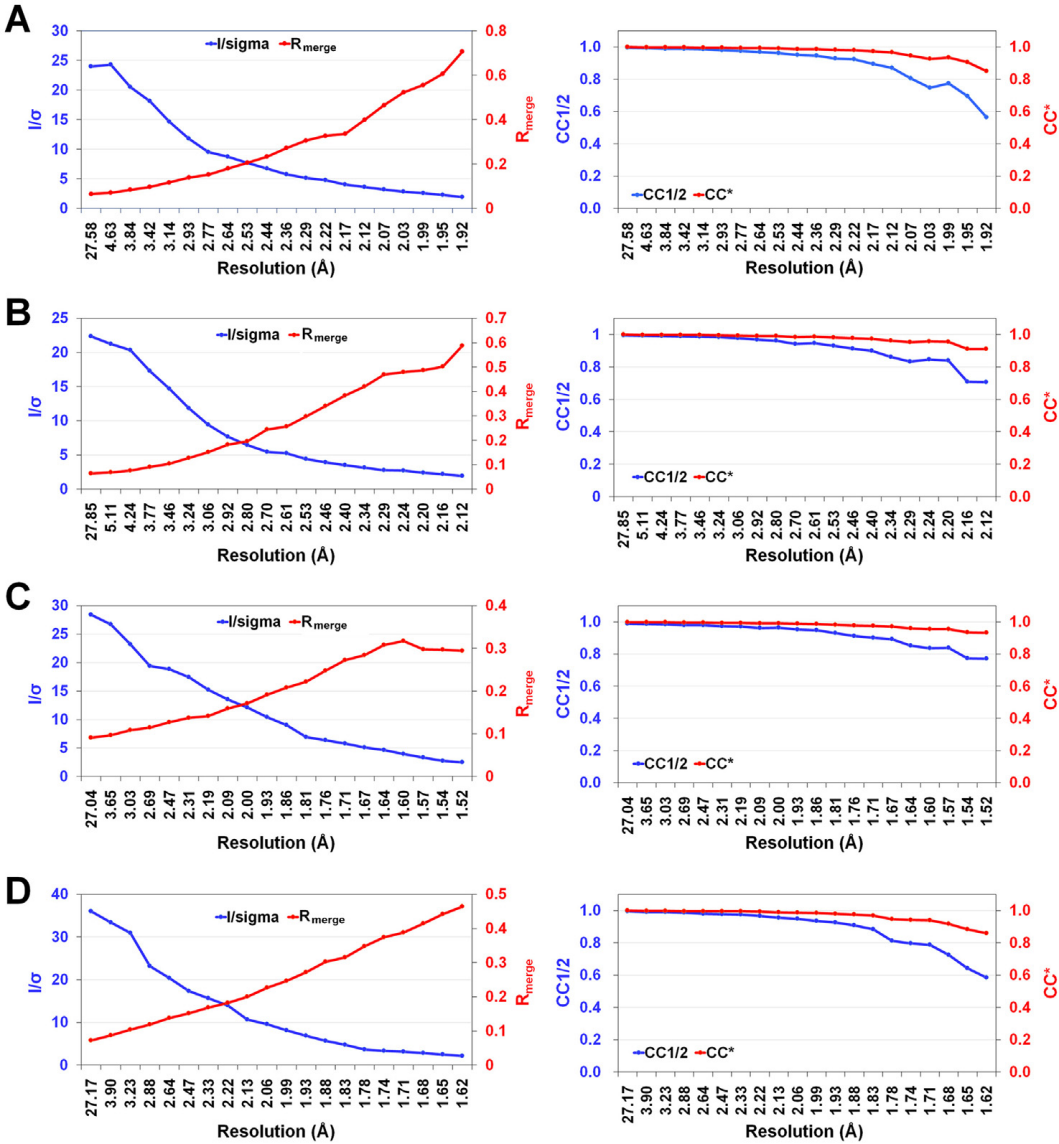
Data processing results for TsaBgl: (A) Data I, (B) Data II, (C) Data III, and (D) Data IV.

Data I were processed to a resolution range of 50.00–1.90 Å, with 123,062 unique reflections. The completeness, redundancy, I/σ(I), R_merge_, CC1/2, and CC* of Data I were 90.7 %, 3.7, 10.78, 0.145, 0.974, and 0.993, respectively (Table 1). For the low-resolution region (50.00–5.16 Å), the completeness, redundancy, I/σ(I), R_merge_, CC1/2, and CC* were 91.7 %, 3.8, 24.0, 0.065, 0.994, and 0.999, respectively (Fig. 1A). In the high-resolution region (1.93–1.90 Å), these values were 91.5 %, 3.4, 1.90, 0.706, 0.563, and 0.849, respectively (Fig. 1A).

**Table 1:** Data processing statistics of TsaBgls.

Data	Data I	Data II	Data III	Data IV
Wavelength (Å)	0.9864	0.9864	0.9864	0.9864
Space group	P1	P1	P2_1_2_1_2_1_	P2_1_2_1_2_1_
Unit cell (Å)				
a	63.41	63.21	64.97	65.06
b	72.83	72.73	70.94	71.21
c	97.49	97.38	98.87	99.15
α	92.49	92.48	90.00	90.00
β	81.28	91.43	90.00	90.00
γ	95.12	95.21	90.00	90.00
Molecule/*asym*.	4	4	1	1
Resolution (Å)	50.00–1.90 (1.93–1.90)	50.00–2.10 (2.14–2.10)	50.00–1.50 (1.53–1.50)	50.00–1.60 (1.63–1.60)
Unique reflections	123,062 (6212)	90,740 (4342)	71,920 (3026)	60,041 (2923)
Completeness (%)	90.7 (91.5)	90.9 (87.2)	97.4 (83.5)	98.8 (97.8)
Redundancy	3.7 (3.4)	3.7 (3.4)	4.1 (2.4)	5.7 (4.6)
Mean/σ(I)	10.78 (1.90)	9.02 (1.92)	15.41 (2.47)	14.63 (2.15)
R_merge_	0.145 (0.706)	0.152 (0.588)	0.130 (0.294)	0.140 (0.464)
R_meas_	0.170 (0.934)	0.177 (0.695)	0.147 (0.358)	0.153 (0.518)
R_pim_	0.087 (0.442)	0.091 (0.368)	0.066 (0.201)	0.059 (0.225)
CC1/2	0.974 (0.563)	0.977 (0.706)	0.973 (0.771)	0.981 (0.585)
CC*	0.993 (0.849)	0.994 (0.910)	0.993 (0.933)	0.995 (0.859)

Values in parentheses are for outer shells.

Data II were processed to a resolution range of 50.00–2.10 Å, with 90,740 unique reflections. The overall completeness, redundancy, I/σ(I), R_merge_, CC1/2, and CC* of Data II were 90.9 %, 3.7, 9.02, 0.152, 0.977, and 0.994, respectively (Table 1). For the low-resolution region (50.00–5.70 Å), the completeness, redundancy, I/σ(I), R_merge_, CC1/2, and CC* were 91.1 %, 3.8, 22.34, 0.064, 0.996, and 0.999, respectively (Fig. 1B). In the high-resolution region (2.14–2.10 Å), these values were 87.2 %, 3.4, 1.92, 0.588, 0.706, and 0.910, respectively (Fig. 1B).

Data III were processed in the resolution range of 50.00–1.50 Å, with 71,920 unique reflections. The overall completeness, redundancy, I/σ(I), R_merge_, CC1/2, and CC* of Data III were 97.4 %, 4.1, 15.41, 0.130, 0.973, and 0.993, respectively (Table 1). For the low-resolution region (50.00–4.07 Å), the completeness, redundancy, I/σ(I), R_merge_, CC1/2, and CC* were 97.4 %, 4.7, 28.44, 0.091, 0.988, and 0.997, respectively (Fig. 1C). In the high-resolution region (1.53–1.50 Å), these values were 83.5 %, 2.4, 2.47, 0.294, 0.771, and 0.933, respectively (Fig. 1C).

Data IV were processed in the resolution range of 50.00–1.60 Å, with 60,041 unique reflections. The overall completeness, redundancy, I/σ(I), R_merge_, CC1/2, and CC* of Data IV were 98.8 %, 5.7, 14.63, 0.140, 0.981, and 0.995, respectively (Table 1). For the low-resolution region (50.00–4.34 Å), the completeness, redundancy, I/σ(I), R_merge_, CC1/2, and CC* were 99.0 %, 6.8, 36.0, 0.072, 0.995, and 0.999, respectively (Table 1D). In the high-resolution region (1.63–1.60 Å), these values were 97.8 %, 4.6, 2.15, 0.464, 0.585, and 0.859, respectively (Fig. 1D).

**Fig. 2:**
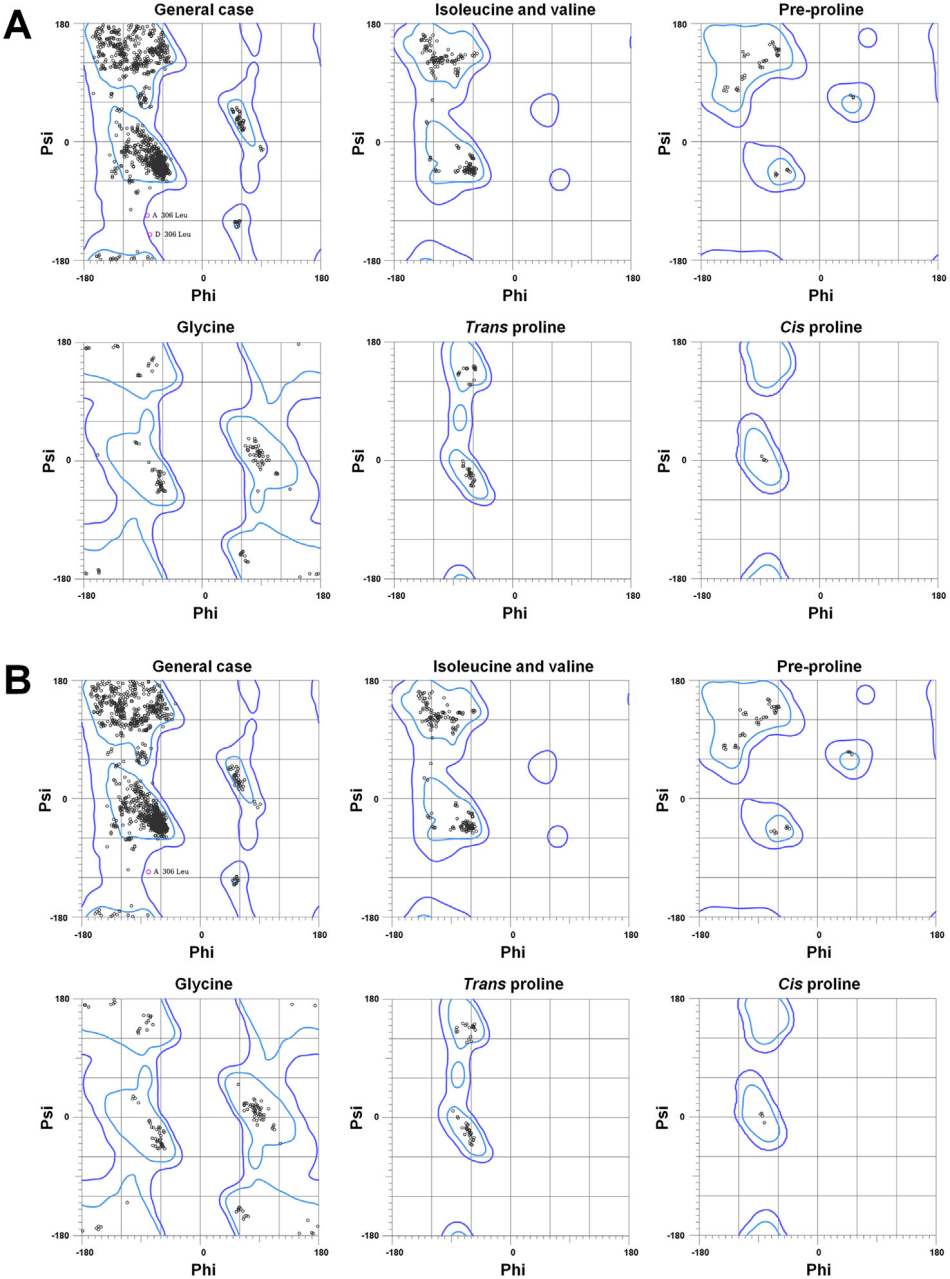
Ramachandran plot of TsaBgl for (A) Data I (PDE code 8WFT) and (B) Data II (8WFU).

The crystal structures of Data I and II, from the P1 space group, were refined to resolutions of 1.9 and 2.1 Å, respectively (Table 2). In the electron density maps for Data I and II, residues of molecules A and B were fully observed. However, the region between residues Gly302 and Gly304 in molecules C and D was partially disordered. The R_work_/R_free_ values for Data I and II were 16.94 %/19.52 % and 15.99 %/20.19 %, respectively. The Ramachandran plots for the final model structures of Data I and II show that 96.99 % and 96.42 % of residues, respectively, were in favored regions, with 0.11 % and 0.06 % in outlier regions (Fig. 2). Specifically, in Data I, Leu306 (Phi and Psi: −84.5° and −112.8°) from molecule A and Leu306 (−80.0° and −141.1°) from molecule D were in the outlier regions. For Data II, Leu306 (−79.5° and −111.2°) from molecule A was in the outlier region.

**Table 2:** Structure refinement statistics for TsaBgl.

Data	Data I	Data II	Data III	Data IV
Resolution (Å)	49.89–1.90	49.81–2.10	49.44–1.50	49.58–1.61
R_work_	0.1694	0.1599	0.1546	0.1506
R_free_	0.1952	0.2019	0.1712	0.1762
RMS deviations				
Bonds (Å)	0.003	0.004	0.003	0.009
Angles (degree)	0.632	0.670	0.648	0.963
B factor (Å^2^)				
Protein	21.99	23.91	12.28	13.40
Water	31.42	31.42	27.10	27.97
Ramachandran plot (%)				
Most favored	96.99	96.48	97.74	97.96
Allowed	2.90	3.46	2.26	2.04
Outliers	0.11	0.06	0.0	0.0
PDB code	8WFT	8WFU	8WFV	8WFW

**Fig. 3:**
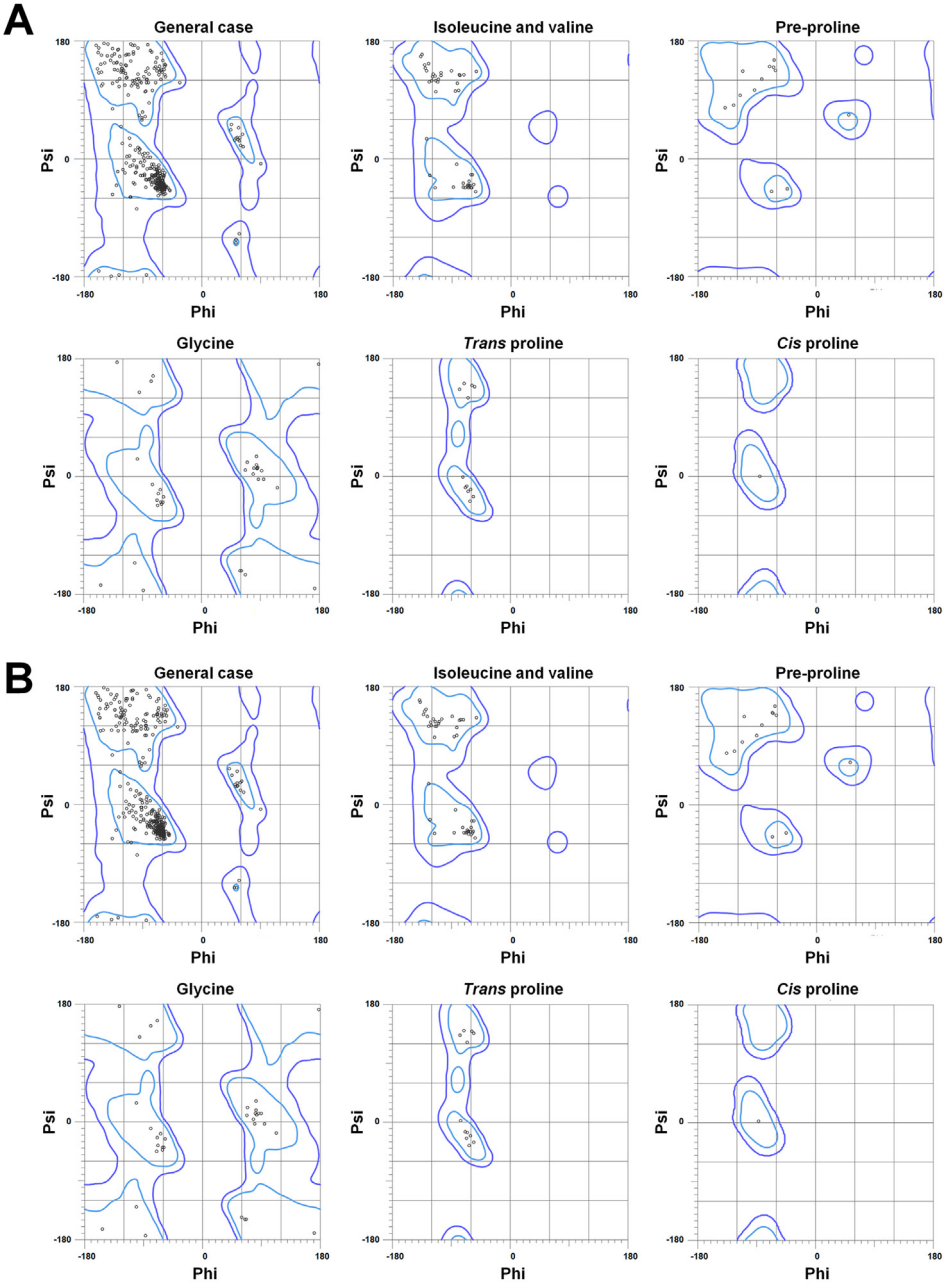
Ramachandran plot of TsaBgl for (A) Data III (PDE code 8WFV) and (B) Data IV (8WFW).

The crystal structures of Data III and IV, from the P2_1_2_1_2_1_ space group, were refined to resolutions of 1.5 and 1.6 Å, respectively (Table 2). The electron density maps allowed for the complete observation and modeling of all amino acid residues. The R_work_/R_free_ values for Data III and IV were 15.46 %/17.12 % and 15.06 %/17.62 %, respectively. The Ramachandran plots for the final model structures of Data III and IV showed that 97.74 % and 97.96 % of residues were in favored regions, with no residues in outlier regions (Fig. 3). The geometry statistics, including poor rotamers, favored rotamers, Ramachandran distribution Z-scores, bad bonds, bad angles, and peptide omega angles, are listed in Table 3.

**Table 3:** Summary of geometry statistics for the four TsaBgl datasets.

	Parameter	Data I	Data II	Data III	Data IV
Protein Geometry	Poor rotamers	10 (0.65 %)	16 (1.04 %)	1 (0.26 %)	1 (0.26 %)
	Favored rotamers	1421 (92.33 %)	1391 (90.09 %)	376 (96.41 %)	372 (95.38 %)
	Ramachandran outliers	2 (0.11 %)	1 (0.06 %)	0 (0.00 %)	0 (0.00 %)
	Ramachandran favored	1708 (96.99 %)	1698 (96.42 %)	432 (97.74 %)	433 (97.96 %)
	Rama distribution Z-score	−0.51 ± 0.18	−0.91 ± 0.18	−0.18 ± 0.37	−0.22 ± 0.37
	Cβ deviations >0.25Å	0 (0.00 %)	0 (0.00 %)	0 (0.00 %)	0 (0.00 %)
	Bad bonds:	0/15,111 (0.00 %)	2/15,131 (0.01 %)	0/3817 (0.00 %)	0/3817 (0.00 %)
	Bad angles:	2/20,431 (0.01 %)	2/20,476 (0.01 %)	1/5169 (0.02 %)	0/5169 (0.00 %)
Peptide Omegas	Cis Prolines:	4/52 (7.69 %)	4/52 (7.69 %)	1/13 (7.69 %)	1/13 (7.69 %)
	Cis nonProlines:	4/1718 (0.23 %)	4/1722 (0.23 %)	1/435 (0.23 %)	1/435 (0.23 %)
Additional validations	Chiral volume outliers	0/2053	0/2059	0/519	0/519

**Fig. 4:**
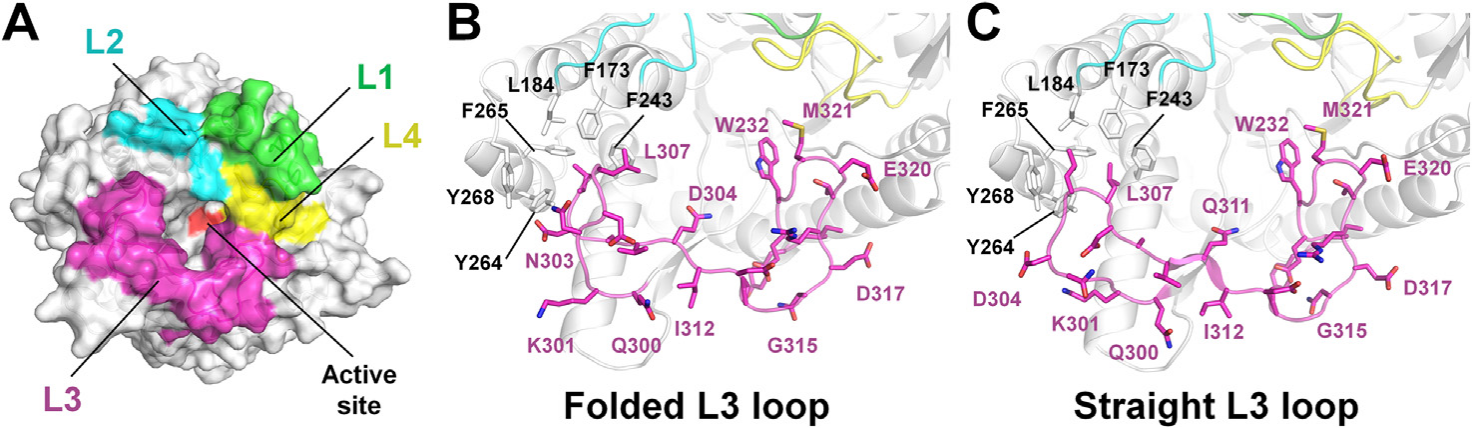
Crystal structure of TsaBgl (PDB code 8WFU). (A) Surface representation of TsaBgl, with the L1–L4 loops colored green, cyan, pink, and yellow, respectively, positioned above the active-site pocket. (B) Structural distinction of the folded L3 loop (chain A). (C) Structural distinction of the straight L3 loop (chain C). (For interpretation of the references to color in this figure legend, the reader is referred to the web version of this article.)

The loops above the substrate-binding pocket in TsaBgl—L1 (Gln39-Asp54), L2 (Gly175-Asp183), L3 (Gln300-Tyr326), and L4 (Trp398-Ile416)—play a role in substrate access to the binding pocket (Fig. 4A). Structural analysis revealed that the conformations of loops L1, L2, and L4 across the four TsaBgl datasets were similar, indicating a structurally rigid conformation. In contrast, the L3 loop exhibited two distinct conformations across the datasets: a folded and a straight conformation (Figs. 4B and C). These conformational variations in the L3 loop affect the substrate-binding site entrance, which can impact substrate accessibility and enzyme activity.

**Fig. 5:**
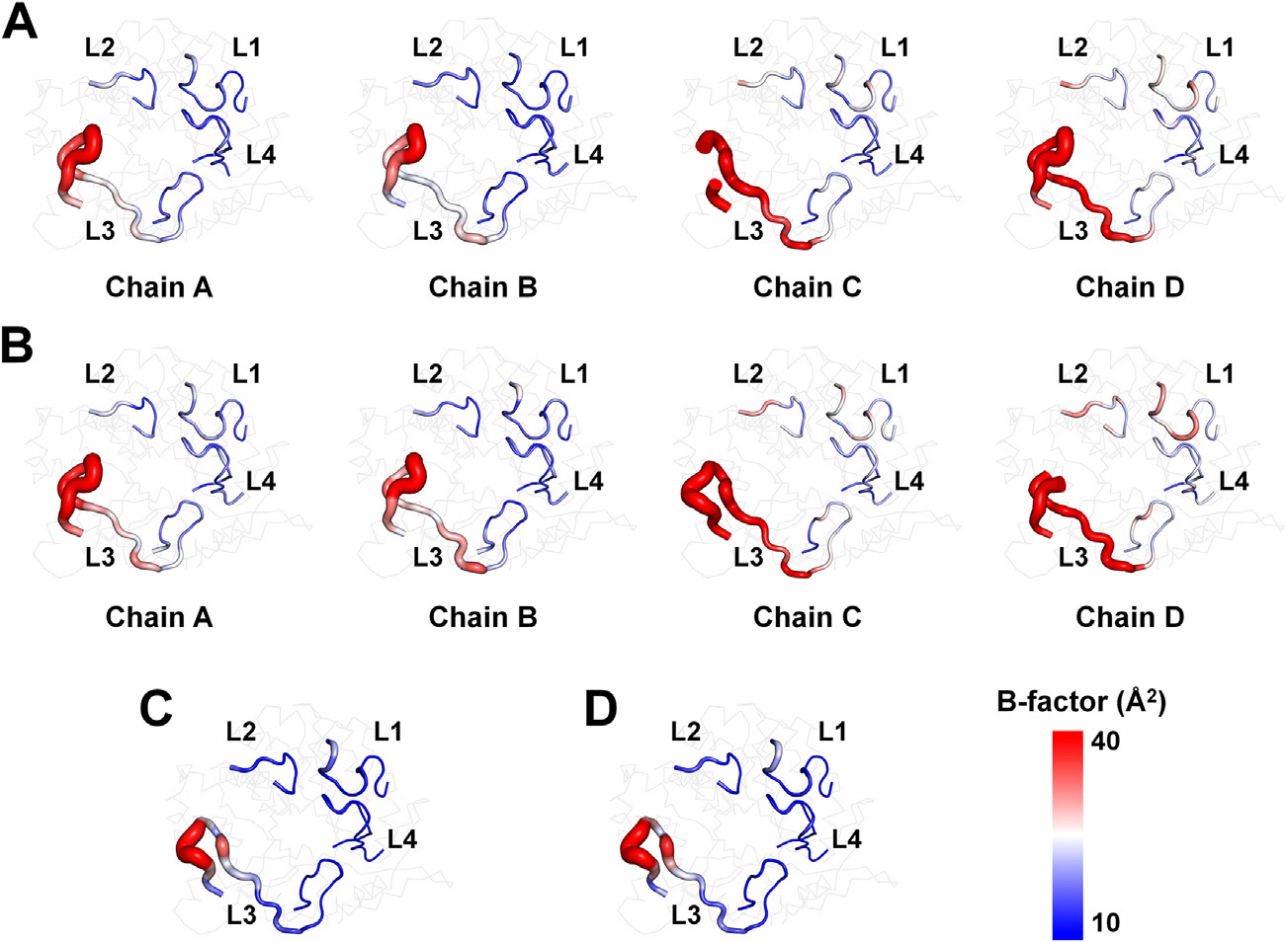
B-factor putty representation of TsaBgl for (A) Data I, (B) Data II, (C) Data III, and (D) Data IV.

The B-factor putty representation shows that TsaBgl L1, L2, and L4 loops exhibit rigid conformations, whereas the L3 loop demonstrates relatively high flexibility (Fig. 5). Particularly, the B-factor values for L1, L2, and L4 loops are lower than the average B-factor of the entire protein, whereas the L3 loop has higher B-factor values than other regions (Table 4).

**Table 4:** B-factor values for the L1–L4 loops in TsaBgl.

Data	Chain	B-factor (Å^2^)				
		Whole protein	L1 loop (Qln39-Asp54)	L2 loop (Gly175-Asp183)	L3 loop (Gln300-Tyr326)	L4 loop (Trp398-Ile416)
Data I	A	20.97	17.31	19.36	27.64	15.36
	B	20.30	15.79	19.62	26.16	14.64
	C	22.90	22.62	19.99	37.74	17.23
	D	24.18	25.81	19.69	38.91	19.92
Data II	A	22.95	19.79	19.21	30.04	17.31
	B	22.21	17.55	19.29	28.79	16.37
	C	25.20	25.74	19.50	43.46	19.89
	D	25.65	27.55	19.04	38.89	21.48
Data III	A	12.33	12.77	18.21	21.13	8.77
Data IV	A	13.48	13.82	18.76	23.47	9.40

All XRD images collected at the synchrotron are deposited at Zenodo (DOI: 10.5281/zenodo.8424379). The diffraction datasets are available as ZIP files containing XRD images (in CBF file format), including data collection information such as X-ray wavelength and detector distance. XRD images can be downloaded without permission. The structure factors and coordinate files are available in the PDB with codes 8WFT (Data I), 8WFU (Data II), 8WFV (Data III), and 8WFW (Data IV). Detailed data collection methods and validation reports are accessible in the PDB. The coordinate files (in PDB format) and structure factor files (in MTZ format) can be downloaded without permission.

## 4 Experimental Design, Materials and Methods

Protein preparation, crystallization, XRD data collection, and structure determination were previously reported [1]. Briefly, the codon-optimized TsaBgl gene (UniProt: I3VXG7) was cloned into the pBT7 vector (Bioneer) and transformed into *Escherichia coli* BL21 (DE3). Cells were cultured in LB broth supplemented with 50 µg/mL ampicillin at 37°C until the OD_600_ reached 0.4–0.8. Recombinant TsaBgl protein expression was induced with 0.5 mM isopropyl-β-d-thiogalactopyranoside and incubated at 18 °C for 18 h. Cells were harvested, lysed by sonication, and the supernatant was purified using Ni-NTA affinity chromatography. The eluted protein was treated with thrombin (Sigma-Aldrich) to remove the N-terminal hexahistidine tag. Further purification was achieved using size-exclusion chromatography on a Sephacryl S-100 column (GE Healthcare) with a buffer containing 10 mM Tris–HCl (pH 8.0) and 200 mM NaCl. The protein was concentrated to 20 mg/mL using a Centricon (10 kDa cutoff; Millipore Merck) for crystallization. Crystals of TsaBgl suitable for XRD experiments were obtained using the hanging-drop vapor diffusion method at 20°C. The crystallization conditions included 0.1 M Tris–HCl (pH 7.5–8.0), 15 %–20 % (w/v) polyethylene glycol 4000, and 0.2 M MgCl. XRD data were collected at Beamline 11C of the Pohang Light Source II (Republic of Korea) [12]. Four XRD datasets were collected using different single crystals. Crystals were cryoprotected by adding 20 % (v/v) ethylene glycol to the crystallization solution and data were collected at 100 K using a Pilatus3 6 M detector. The detector-to-crystal distance for all datasets was 300 mm. The XRD data were processed with the HKL2000 program [13]. The horizontal and vertical box sizes for processing the diffraction peaks were both set to 4.128 for all four datasets. The phase problem was solved by molecular replacement using the MOLREP program [14] with the TsaBgl crystal structure (PDB code 7E5J) [15] as the search model. Structure building was conducted with the Coot program [16]. Structure refinement was performed using the refine.phenix function in the PHENIX program [17]. The geometry of the model structures was validated using MolProbity [18].

## Limitations

Not applicable.

## Ethics Statement

This work meets the ethical requirements for publication in this journal. This work does not involve human subjects, animal experiments, or any data collected from social media.

## CRediT Author Statement

**Ki Hyun Nam**: Conceptualization, methodology, formal analysis, investigation, writing—original draft preparation.

## Data Availability

ZenodoX-ray diffraction images (Original data). Protein Data BankCrystal structure of β-glucosidase from Thermoanaerobacterium saccharolyticum (Data I) (Original data). Protein Data BankCrystal structure of β-glucosidase from Thermoanaerobacterium saccharolyticum (Data II) (Original data). Protein Data BankCrystal structure of β-glucosidase from Thermoanaerobacterium saccharolyticum (Data III) (Original data). Protein Data BankCrystal structure of β-glucosidase from Thermoanaerobacterium saccharolyticum (Data IV) (Original data).
